# Medical and Physical Intelligence

**Published:** 1823-03

**Authors:** 


### MEDICAL AND PHYSICAL INTELLIGENCE.

### 1. *Some Account of the Medical Schools, &c. at Berlin.—(Extract of a Letter to* Dr. Granville, *from a Physician at Berlin.)*

[Concluded from page 173.]

The accoucheur resides in the hospital, as well as the assistants, and the patients receive all the advantages that could be afforded in a private house. There is a cabinet furnished, as usual, with every instrument, ancient and modern,—with preparations in wax and spirits, and dolls of all descriptions,—but, above all, with excellent representations of the neck of the uterus in various stages of gestation, up to the moment of delivery: these are composed of elastic gum; there are forty pieces in all, which, with the box that contains them, cost thirty-six dollars. They are made at Potsdam, by a person who has a patent. Every birth is attended by young men, as in the private houses in Paris, and even girls may have instruction, if they wish for it. In short, the treatment is excellent; the patients are well nourished, are made to work when they are able, and nothing is wanting but a garden for the convalescents. In peritonitis, they are very active in the application of leeches, ipecacuanha, and calomel; and in this department, indeed, it does not appear to me that there is any thing to desire, and therefore it is not wonderful that they should mention their lying-in establishment, when they boast their superiority over England, which on this occasion must certainly concede it to them.

The deaf and dumb in Berlin understand, and make themselves understood, better than those of London: they are in constant communication with those who have the use of their faculties, and are able to express themselves tolerably well. It is to be observed, that the German language is better adapted than the English for the speech of the deaf, because this latter is more deficient in gutturals, and because the lips perform the principal part in the pronunciation. In one word, they are not deaf, for they hear with their eyes; they are not dumb, for they articulate sounds in such a manner as to be understood. Their education, in other respects, is the same as in other countries; and here, as well as in other points, they consider themselves superior to all others.

The Hospital for the Blind is not a place of labour, as in London, but of a much more refined sort of education, and therefore similar to that of Paris. They are taught music, by means of notes cut in wood; they are taken to the museum, in order to teach them to distinguish animals by the touch; they are also taught the points of the compass, and become proficients in geography, which they learn on large maps with the places in relief; they learn to count with small square pieces of wood, or with the Russian chain, as it is called, consisting of some dozens of little balls strung on an iron wire. Geometrical figures are also presented to them in wooden models.

The Veterinary School is as extensive as that of Paris; nay, indeed, it is perhaps larger. What this establishment loses in comparison with the school at St. Pancras, from the superiority in the construction of the stalls in this last, it regains on account of the greater commodiousness of the amphitheatre, baths, &c. The amphitheatre contains a good collection of anatomical and pathological preparations; and there is a peculiarity in the construction of the table, which, (independently of its turning upon its axis, by a particular mechanism situated under ground,) sinks below the floor, and comes up charged with the dead body. The bath for the horses is magnificent and useful; but they do not here practise the excision of the nerve, as in certain cases of lameness in London. I fancy by this time I must have exhausted your patience; but I have not yet quite done: let me indulge in a few general remarks, and then I will put down my pen.

Foreign languages are very generally understood in this country: this gives them a high opinion of themselves, and induces them to believe that Germany is the most literary country in the world; but they attach a very important meaning to that expression. A medical literary man is one who pays great attention to the languages, and to the progress of the sciences in foreign countries; and there are many persons, young men especially, who have travelled, who do nothing more than make extracts from new foreign publications, and send them to some journalist, who pays them, and puts their names to the work, if they wish it. Is not this the real reason that Germany and its journals are so deficient in originality? Is not this confessing tacitly, and as it were in despite of themselves, the superiority of other nations? Here every professor is editor of a medical Journal: thus, Hufeland has his; Gräfe and Walther publish a Journal of Surgery and Ophthalmalogia, and this possesses more originality than the others; Rust has his Magazine, and Dr. Horn and Professor Wagner publish another. * * I have already said that the Germans are well acquainted with foreign languages, but they very often know but little of their own: attracted by the charms of the French language, they generally study it from their earliest youth, and thus they make a strange mixture in their compositions.

* * * *

There are only two things that I have to regret in leaving Berlin; the first is the agreeable society of Rudolphi, and the second is the pleasure of reading all the literary Journals in the Professor's Cabinet at the Royal Library, and the English papers at the Literary Cabinet at the Exchange, where 180 papers of various nations are to be found. These two establishments are not to be rivalled in any part of Europe, or perhaps in the world. In the first, public and private professors only are admitted; in the last, the introduction of a member gives the privilege of admittance, paying one dollar a month,—a trifle, considering the nature of the institution.

---

### 2. *Experiments on the Cerebellum and Cerebrum.*

1st. M. Flourens removed the cerebellum in successive layers from a pigeon. At the taking away of the first slice, the animal experienced but little weakness and hesitation in its motions. At the middle layers, its walk became unsteady and agitated, altogether like that of a drunken person; soon it could not walk without the assistance of its wings. The sections being continued, the animal lost altogether the faculty of walking; its feet were no longer sufficient to support it, and it had to sustain itself on its tail and wings: it often attempted to walk or fly, but always without success. If it was pushed forward, it tumbled over its head; if backwards, it rolled on its tail. The sections were carried farther: the animal then lost the faculty of keeping itself up on its wings and its tail; it tumbled continually, without being able to stop in any fixed position, or it finally rested flat on its back or belly. In other respects, it saw and heard very well; its air was lively, its head erect, and spirited.

2d. M. Flourens removed from a pigeon the right cerebral lobe: the animal lost instantly the sight of the left eye; but the contractility of the iris of that eye continued unchanged. There was also a marked feebleness in the right side of its body. With the exception of these two circumstances, the animal was well: it sustained itself, walked, ran, flew, saw with the other eye, understood, wished, felt as usual. The other lobe being removed, the sight of both eyes was instantly lost, but not the contractility of the iris. There was at first a very distinct general debility: otherwise, the animal held itself perfectly upright on its feet; and, in whatever position they put it, it maintained its equilibrium. It walked, when pushed; it flew, when tossed into the air; but, left to itself, it remained plunged as it were in a continual stupor. It never moved, except in proportion as it was irritated; it gave no sign of volition. Memory, vision, hearing, will, all its perceptions, were extinguished. There is none of his numerous experiments which M. Flourens has not repeated on each of the four classes of vertebrated animals; and he has always indicated the shades of greater or less depth which characterize these classes.—(*Journal of Science.*)

### 3. *On the Use of Iodine.*

Iodine has for some time past been frequently employed by the medical practitioners of this place, particularly among the inhabitants of a mountainous district of country, situated a few miles from Cologne, where a considerable number of cases of goitre are met with. I have, along with others, tried its medicinal effects, particularly in the cases of two sisters, the one sixteen, the other eighteen years old; the former of whom was affected with a small tumor of the bronchocele kind, about the size of a pigeon's egg, situated in the neighbourhood of the thyroid gland; the other had a chronic tumor, of much greater magnitude, situated on the left side of the neck: the swellings in both instances were hard, and quite free from pain. After the use of the iodine for a fortnight, considerable diminution in the size of the tumors was already observed. During that time the patients had only employed the Ungt. Hydriodin. made thus:—℞. Axung. porc. ℥jss. et Kali Hydriodinicum, ʒss. M. et fiat ungt. Of this ointment, about the size of a hazel-nut was rubbed-in on the swelling every evening; the iodine not being at all exhibited internally. At the present moment, they still continue the use of the ointment; and I do not in the slightest degree doubt but that the tumors will be discussed without leaving the smallest trace of them, since they are already of very inconsiderable size. During the employment of this application, the patients were not affected with any particular symptom, with the exception of the younger of the two, who, about the sixth day after she began the application, complained of slight pains in the tumor; but these entirely disappeared in two days afterwards, the use of the ointment remaining uninterrupted. Since then they have not returned.—(*Extract of a Letter from* Dr. GUNTHER, *of Cologne, to the Editor of the Medicinisch-Chirurgische Zeitung;* 1st August, 1822.)

---

### 4. *Test for Oxalic Acid.*

IN consequence of the numerous accidents which have occurred from mistaking oxalic acid for Epsom salts, several tests have been proposed to distinguish them. Tasting is certainly the readiest method; but few people like to taste a reputed poison. Test-papers, prepared by turmeric or by litmus, do very well, but are not always at hand. A more ready method would be to take a little common ink in a writing-pen, and drop into it one or two of the crystals of the suspected salts: if it be Epsom salts, the ink will remain unchanged in colour; if it be oxalic acid, the ink will become of a light reddish brown, and no longer appear as ink, the acid dissolving the black oxide, and forming oxalate of iron. It may be remarked that oxalic acid is only poisonous when taken in doses of from half an ounce to two ounces: small quantities of it are not deleterious. The writer of this notice, while a student of medicine, was in the habit of acidulating water with it for his common drink, and never experienced any bad effects from its use.—(*Quarterly Journal of Foreign Science.)*

---

### 5. *Cases of Puerperal Convulsions.* By Dr. ALPHONSE MENARD.

A YOUNG woman, between nineteen and twenty years of age, of good constitution and very stout, in her first pregnancy, had been in labour four days, when I was called to attend her. On my arrival, she was in dreadful convulsions affecting the whole body; the features, particularly, were horribly distorted; the tongue clenched between her jaws, foaming at the mouth, &c. She remained in this state nearly a quarter of an hour, which was succeeded by an universal torpor of the like duration, when she became sensible, but without the slightest recollection of what had happened. It was then about one in the afternoon. Finding the pulse full and rapid, I took a large quantity of blood from the arm, and prescribed a mixture containing the vitriolic æther and ammonia. The orifice of the uterus was rigid, hot, and half dilated; and the head of the child presenting appeared to be very large. She had no flooding nor discharge of any kind. The abdomen exhibited two distinct convex surfaces; one, produced by the distended uterus, occupied the space extending from the epigastrium to the umbilical cicatrix; the other, immediately above the pubis, caused by the bladder, which was distended with urine. The labour-pains, which at first were strong, for the last hour had scarcely been sensible. After having fulfilled the first indication by bleeding, it remained to draw off the water, and hasten the delivery, as the distention of the womb appeared to be the cause of the convulsions. I attempted every method of introducing the catheter, without success. I then turned all my attention to the os uteri, but was interrupted by another fit, which however was not of so long duration as the preceding one. A small wooden cylinder was inserted betwixt the teeth. Without entering into particulars, I shall merely state, that the dilatation of the mouth of the uterus was exceedingly tedious and painful, during which time the patient had six attacks of similar fits, though not equal in duration: the contraction of the jaws, in one instance, was so strong, that two of the incisor teeth were broken. Venesection was repeated three times, and a warm bath administered, in which she could only remain half an hour. At midnight, during the last fit, I was able to introduce my hand into the uterus; during the succeeding collapse, I was enabled to empty the bladder, turn and extract a stout male child, which was dead. Its death, I suppose, happened about the time of the first convulsion; and, from the contraction of the extremities (the inferior ones particularly,) I presume it had sympathised with the mother,—a circumstance which struck the assistants as well as myself. Another circumstance deserving notice is, that the lochial discharge did not take place till twelve hours after the accouchement. The subject of this observation experienced no accident after the expulsion of the fœtus and secundines, and enjoys perfect health at present.

* * * *

During my residence in Paris, in 1820, I was called to attend a lady, who had been in labour two days at the time I was sent for, and affected by convulsions similar to those described above. Every symptom indicated a general plethora, and I immediately let blood to a large extent. The pains recurred within a quarter of an hour after the depletion, and the left thigh of the patient (to use her own expression) was benumbed from excess of pain. On examination, I found that the os uteri was concealed in the left iliac fossa, with the head of the child pressing strongly above it: this I considered as the cause of the mischief. I proceeded to lay the patient on the right side, and, pressing the abdomen with one hand, used the other in dilating and drawing the orifice of the uterus to its proper situation; after which, with considerable difficulty, I seized the child by the legs, and effected the delivery. The fits in this case also recurred at irregular intervals. The following day, the patient had no recollection of having been delivered, was without fever, and recovered rapidly; since which time she has had a child (now living,) without any accident. In this, as well as in every other case I have observed, the child was born dead; and the contraction of the extremities and features evidently denote its having participated in the affection of the mother. The woman attacked by convulsions has no warning of the return of the paroxysm, but I have noticed its approach by particular signs: thus, in the first case, the patient experienced an intense thirst previous to the first three attacks, and the four last were preceded by feeble but repeated pains. In the last case, a convulsive action of the eyeball, and a tetanic affection of the wrist, was perceptible. Lastly, the pulse, in all cases I have seen, gave the most certain indication: at the commencement it was full and quick, then gradually got weaker during the paroxysm, still retaining its rapidity; at the termination it gradually diminished, till almost imperceptible, then progressively regained its vigour as the patient recovered her faculties.—(*Journal Complémentaire*, Janvier 1823.)

### 6. *Vaccination in the Roman States.*

By a late edict which has been published, at Rome, by the Papal secretary of state, it appears that very important arrangements, in various points of view, have been made towards the more general and perfect propagation of vaccination. A central establishment, with a secretary, &c. has already been organized; and soon a vaccinating commission will be appointed for each of the provinces. (*Vienna Gaz.*)

### 7. *Small-Pox in Prussia.*

In the Prussian monarchy, out of 484,523 children, born in the year 1821, 40,000 have been vaccinated. During the above period, there died of small-pox, in all the provinces under the dominion of the King of Prussia, 1,190 persons. Before the introduction of vaccination, from 30 to 40,000 individuals annually died of the small-pox. (*Ibid.*)

### 8. *Faculty of Medicine in Paris.*

The Faculty of Medicine of Paris, which had been suspended, has just been restored by an ordinance of the King, but with certain changes and modifications. There are to be twenty-three Professors and a certain number of Fellows: only these last will be present at meetings, and will have the privilege of giving private courses. Many of the old Professors will retain the title of *Honoraries*.—(*Private letter, dated February 5,* 1823.)

### 9. *Medical Botany.*

We have frequently had occasion to regret the neglect of due attention to the study of Botany in our profession; we are therefore gratified to find that Mr. Frost, the director of the Medico-Botanical Society, is about to publish, in the form of a Journal, such improvements or discoveries as are occasionally made in medical botany and pharmacy. We trust the undertaking will meet with the encouragement which the acquirements of its conductor lead us to believe it will merit.

### 10. *Detection of Poisons.*

A paragraph has appeared in the papers, recommending blue sugar-loaf paper as a test of distinction between oxalic acid and Epsom salt: it is reddened by the former, but not affected by the latter. This is perfectly true; but a simpler test consists in wetting the tip of the finger, applying it first to the supposed salt, and then to the tongue: if oxalic acid, it tastes very sour; if Epsom salt, very bitter and saline.

Many restrictions have been suggested upon the sale of arsenic; the only effectual bar to the mischief that results from it, is prohibition. It is of no use in medicine, and should therefore be rejected from the Pharmacopœia: apothecaries and druggists need not then keep it. It is sometimes employed as an instrument of research, in the chemical laboratory, which might be sufficiently supplied through other channels. —*(Journal of Science.)*

### 11. BILLS OF MORTALITY,

*From December* 12, 1821, *to December* 11, 1822.

| DISEASES. | |
|---|---|
| Abscess | 107 |
| Apoplexy | 206 |
| Asthma | 533 |
| Bedridden | 1 |
| Cancer | 82 |
| Childbed | 191 |
| Consumption | 3608 |
| Convulsions | 2929 |
| Croup | 100 |
| Diabetes | 3 |
| Dropsy | 851 |
| Dropsy in the Brain | 324 |
| Dropsy in the Chest | 86 |
| Dysentery | 4 |
| Epilepsy | 2 |
| Eruptive Diseases | 6 |
| Erysipelas, or Saint Anthony's Fire | 17 |
| Fever | 1104 |
| Fever (Typhus) | 17 |
| Fistula | 6 |
| Flux | 6 |
| Gout | 41 |
| Hæmorrhage | 31 |
| Hooping-Cough | 757 |
| Inflammation | 1308 |
| Inflammation of the Liver | 61 |
| Insanity | 218 |
| Jaundice | 111 |
| Measles | 712 |
| Mortification | 159 |
| Old Age, and Debility | 2601 |
| Palsy | 169 |
| Rheumatism | 8 |
| Rupture | 44 |
| Scrofula | 7 |
| Small-pox | 604 |
| Sore Throat or Quinsey | 5 |
| Spasm | 55 |
| Still-born | 667 |
| Stone | 16 |
| Stoppage in the Stomach | 16 |
| Suddenly | 220 |
| Teething | 472 |
| Thrush | 102 |
| Venereal | 7 |
| Worms | 3 |
| Total of Diseases | 18577 |

| CASUALTIES. | |
|---|---|
| Broken Limbs | 1 |
| Burnt | 18 |
| Drowned | 113 |
| Excessive Drinking | 4 |
| Executed* | 8 |
| Found Dead | 6 |
| Fractured | 2 |
| Killed by Falls and several other Accidents | 84 |
| Murdered | 4 |
| Overlaid | 1 |
| Poisoned | 3 |
| Scalded | 7 |
| Suicide | 33 |
| Strangled | 1 |
| Suffocated | 3 |
| Total of Casualties | 288 |

| CHRISTENED. | | | BURIED. | | |
|---|---|---|---|---|---|
| Males | 11968 | In all, 23373 | Males | 9483 | In all, 18865 |
| Females | 11405 | | Females | 9382 | |

Whereof have died,

| | | | |
|---|---|---|---|
| Under Two Years of Age | 4605 | Sixty and Seventy | 1562 |
| Between Two and Five | 2033 | Seventy and Eighty | 1224 |
| Five and Ten | 932 | Eighty and Ninety | 680 |
| Ten and Twenty | 649 | Ninety and a Hundred | 104 |
| Twenty and Thirty | 1348 | A Hundred | 1 |
| Thirty and Forty | 1905 | A Hundred and One | 1 |
| Forty and Fifty | 1995 | A Hundred and Two | |
| Fifty and Sixty | 1826 | A Hundred and Eight | |

Increased in the Burials this Year, 414.

### 12. *Population of Russia, and Instances of Longevity.* (From the Edinburgh Philosophical Journal, No. XV.)

In the year 1817, the number of births in Russia is stated at 786,810 boys, and 711,796 girls; the number of deaths at 423,092 males, and 405,469 females, of whom 208,954 died under five years

* There have been executed in London and the county of Surrey, 24; of which number, 8 only have been reported to be buried within the Bills of Mortality.

of age. The increase of population was 670,045. The number of individuals who had attained the age of

| | | | | | |
|---|---|---|---|---|---|
| 60 years was | | 68,723 | 125 years was | | 21 |
| 70 | — | 38,765 | 130 | — | 17 |
| 80 | — | 16,175 | 135 | — | 1 |
| 90 | — | 2,108 | 140 | — | 1 |
| 100 | — | 783 | | | |
| 115 | — | 83 | | | |
| 120 | — | 51 | | | |

Total, 126,728; which is about 1-7th part of the deaths.

### METEOROLOGICAL JOURNAL.

By Messrs. William Harris and Co. 50, Holborn, London.

*From January* 20, 1823, *to February* 19, 1823.

| | MOON. | Rain gauge | THERM. | | | BAROM. | | DE LUC'S HYG. | | WIND. | | ATMOSPHERIC VARIATION. | | |
|---|---|---|---|---|---|---|---|---|---|---|---|---|---|---|
| | | | 9 A.M. | MAX. | MIN. | 9 A.M. | 10P.M. | 9 A.M. | 10P.M. | 9 A.M. | 10P.M. | 9 A.M. | 2 P.M. | 10P.M. |
| Jan. 20 | ☾ | | 16 | 26 | 30 | 29.56 | 29.61 | 84 | 90 | E | ENE | Foggy | Fine | Snow |
| 21 | | | 27 | 30 | 24 | 29.70 | 29.77 | 87 | 89 | E | E | Cloudy | | |
| 22 | | | 25 | 26 | 20 | 29.80 | 29.82 | 82 | 80 | E | E | Fine | Cloud. | |
| 23 | | | 22 | 29 | 22 | 29.70 | 29.53 | 86 | 80 | ESE | ESE | Cloudy | Fine | |
| 24 | | | 24 | 25 | 22 | 29.60 | 29.73 | 75 | 85 | E | E var. | Fair | Fine | |
| 25 | | | 23 | 24 | 21 | 29.68 | 29.52 | 80 | 96 | E | ESE | Fair | Snow | |
| 26 | ○ | | 26 | 30 | 28 | 29.63 | 29.65 | 90 | 94 | ENE | E | Fair | | |
| 27 | | | 29 | 31 | 36 | 29.52 | 29.33 | 84 | 98 | SE | SSE | Cloudy | Rain | |
| 28 | | | 40 | 44 | 40 | 29.34 | 29.20 | 100 | 95 | SW | SW | Rain | Cloud. | Rain |
| 29 | | | 45 | 49 | 44 | 29.02 | 29.02 | 99 | 98 | SW | W | Rain | Sho'ry | Cloud. |
| 30 | | | 45 | 44 | 40 | 29.30 | 29.31 | 98 | 96 | W | WSW | Cloudy | Fine | Fair |
| 31 | | | 41 | 41 | 39 | 29.06 | 28.90 | 97 | 78 | ESE | ESE | Cloudy | | Rain |
| Feb. 1 | | | 40 | 45 | 34 | 28.75 | 28.64 | 79 | 98 | E | E | Rain | | |
| 2 | ☽ | .43 | 38 | 42 | 40 | 28.55 | 28.63 | 99 | 98 | ENE | E | Rain | | |
| 3 | | .17 | 41 | 43 | 30 | 28.77 | 29.00 | 99 | 94 | E | NW | Foggy | Rain | |
| 4 | | | 33 | 37 | 29 | 29.20 | 29.27 | 83 | 76 | WNW | WNW | Fine | | |
| 5 | | | 30 | 35 | 30 | 29.35 | 29.63 | 76 | 81 | NW | ENE | Fine | | |
| 6 | | | 31 | 34 | 30 | 29.37 | 29.25 | 88 | 97 | SE | SE | Fine | Snow | |
| 7 | | .29 | 32 | 36 | 31 | 29.17 | 29.00 | 100 | 95 | S | SSE | Rain | | |
| 8 | | | 33 | 39 | 33 | 29.34 | 29.40 | 85 | 79 | WNW | WSW | Fine | | |
| 9 | | .11 | 37 | 42 | 37 | 29.51 | 29.34 | 76 | 88 | NW | W var. | Very fine | | Rain |
| 10 | | .15 | 42 | 48 | 41 | 29.33 | 29.23 | 85 | 88 | WSW | NW | Rain | | Cloud. |
| 11 | ● | .16 | 45 | 51 | 45 | 29.30 | 29.26 | 87 | 89 | WSW | W var. | Rain | | Cloud. |
| 12 | | | 47 | 52 | 39 | 29.21 | 29.20 | 84 | 85 | WSW | WSW | Cloudy | Fine | |
| 13 | | | 41 | 45 | 35 | 29.38 | 29.46 | 71 | 74 | SW | S | Fine | | |
| 14 | | .09 | 37 | 42 | 37 | 29.30 | 29.40 | 77 | 79 | WSW | WSW | Fine | Rain | Cloud. |
| 15 | | .25 | 40 | 44 | 33 | 29.63 | 29.87 | 83 | 87 | NNW | N | Rain | | Cloud. |
| 16 | | | 35 | 39 | 32 | 30.00 | 30.09 | 79 | 83 | NE | NE | Showery | | |
| 17 | | | 34 | 38 | 35 | 30.01 | 29.85 | 85 | 82 | NE | S | Showery | | Fair |
| 18 | ☾ | | 37 | 43 | 35 | 29.64 | 29.21 | 80 | 84 | SE | S var. | Showery | | Rain |
| 19 | | .18 | 37 | 41 | 36 | 29.30 | 29.38 | 79 | 74 | SW | W | Fine | Fair | |

